# Supplementary material for: Gravitational and Dynamic Components of Muscle Torque Underlie Tonic and Phasic Muscle Activity during Goal-Directed Reaching
Source: Front Hum Neurosci. 2017 Sep 26;11:474. doi: 10.3389/fnhum.2017.00474 (PMC5623018; doi:10.3389/fnhum.2017.00474)
Supplement: Supplementary file 1 [file Image1.pdf]

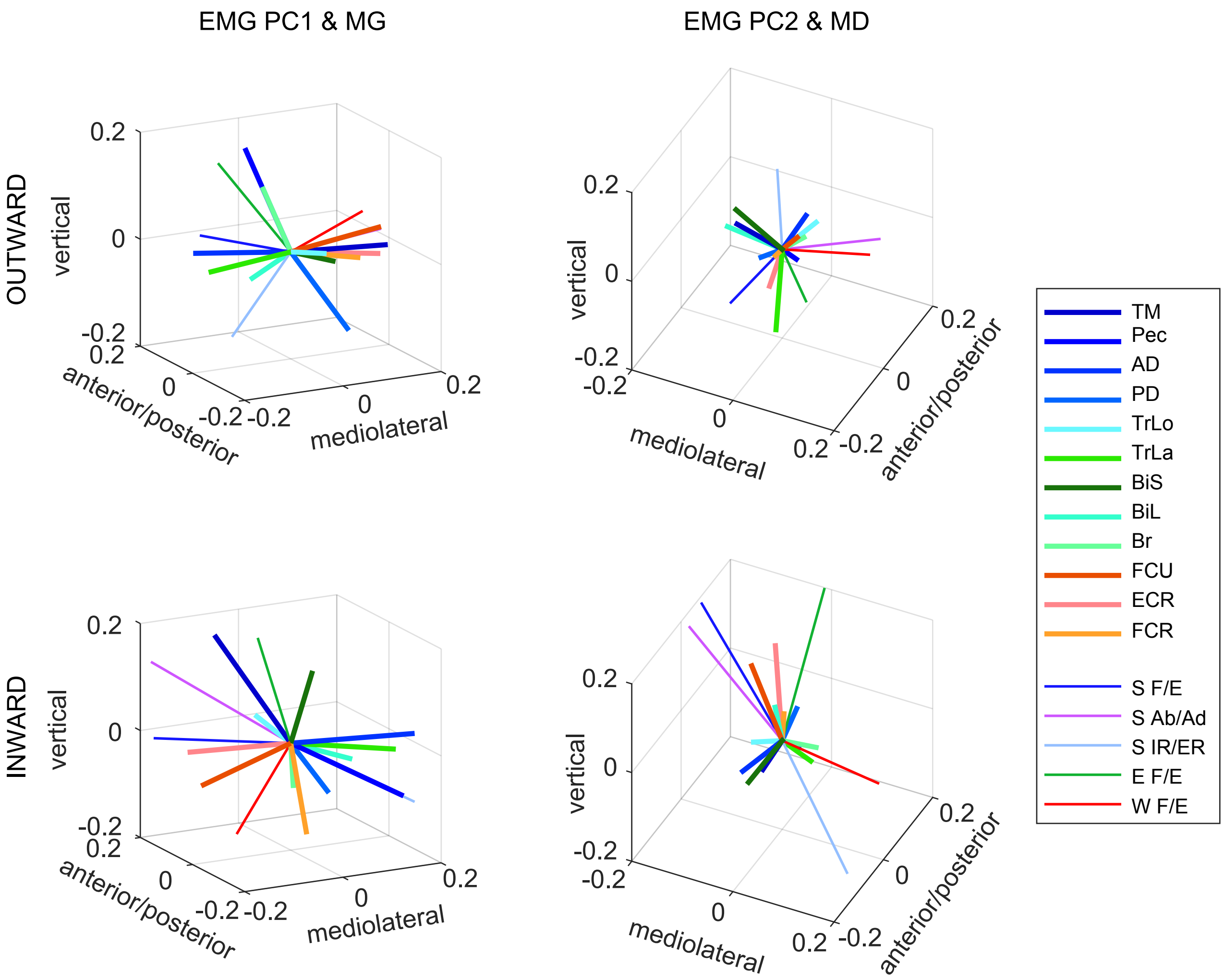

Supplementary figure. Directional tuning of the eigenvalues from torque PCA and z-scores from EMG decomposition. For torque PCA, only the first principal component (PC) was included for MG and MD. For EMG decomposition, the first 2 principal components were included. The sign of eigenvalues and z-scores was controlled to ensure that the waveforms of PCs did not flip. The eigenvalues and z-scores determine the amplitude of each plotted vector, the target locations determine their Cartesian coordinates. Horizontal targets 1-8 were in the anterior/posterior and mediolateral plane, while the vertical targets 9-14 were in the vertical plane (Fig. 1). Population vectors are plotted in color lines, thick lines for EMG and thin lines for torques. Projections on the Cartesian axes were averaged across subjects, then across movement directions to calculate a population vector for each signal. Plots in rows show data for center-out (outward) and return (inward) movements. Columns show different principal components as specified in the titles. The directional tuning of the population vectors for the 1st gravitational principal component tended to be distinct from that of the second dynamic principal component. There was no obvious correspondence between the directional tuning of EMG and torque components.
